# Digital Pain Drawings Can Improve Doctors’ Understanding of Acute Pain Patients: Survey and Pain Drawing Analysis

**DOI:** 10.2196/11412

**Published:** 2019-01-10

**Authors:** Nour Shaballout, Anas Aloumar, Till-Ansgar Neubert, Martin Dusch, Florian Beissner

**Affiliations:** 1 Somatosensory and Autonomic Therapy Research Institute for Diagnostic and Interventional Neuroradiology Hannover Medical School Hannover Germany; 2 Section Pain Medicine Clinic of Anaesthesiology and Intensive Care Medicine Hannover Medical School Hannover Germany

**Keywords:** pain drawing, symptom drawing, manikins, tablet computers, eHealth, app, acute pain

## Abstract

**Background:**

Pain drawings (PDs) are an important tool to evaluate, communicate, and objectify pain. In the past few years, there has been a shift toward tablet-based acquisition of PDs, and several studies have been conducted to test the usefulness, reliability, and repeatability of electronic PDs. However, to our knowledge, no study has investigated the potential role of electronic PDs in the clinical assessment and treatment of inpatients in acute pain situations.

**Objective:**

The aim of this study was to evaluate whether knowledge of the patients’ electronic PD has the potential to improve the doctors’ understanding of their patients and to influence their clinical decision making. Furthermore, we sought to identify differences between electronic PDs of patients and their treating pain specialists in an acute pain situation and to find those specific characteristics derived from the PDs that had the largest impact on doctors’ understanding.

**Methods:**

We obtained electronic PDs from 47 inpatients in acute pain situations before their consultation with a pain specialist on a tablet personal computer with a stylus. Before looking at their patients’ drawings, these specialists drew their own conception of the patients’ pain after anamnesis and physical examination. Patients’ drawings were then revealed to the doctors, and they were asked to evaluate how much the additional information improved their understanding of the case and how much it influenced their clinical decision on an 11-point Likert scale (0=“not at all” and 10=“very much”). Similarities and differences of patients’ and doctors’ PDs were assessed by visual inspection and by calculating Jaccard index and intraclass correlation coefficient (ICC) of the pain area and the number of pain clusters. Exploratory analyses were conducted by means of correlation tables to identify specific factors that influenced doctors’ understanding.

**Results:**

Patients’ PDs significantly improved the doctors’ understanding (mean score 4.81, SD 2.60, *P*<.001) and to a lesser extent their clinical decision (mean 2.68, SD 1.18, *P*<.001). Electronic PDs of patients and doctors showed fair to good similarity for pain extent (*r*=.454, *P*=.001) and widespreadness (*P*=.447, *r*=.002) were important factors helping doctors to understand their patients.

**Conclusions:**

In a clinical setting, electronic PDs can improve doctors’ understanding of patients in acute pain situations. The ability of electronic PDs to visualize differences between doctors’ and patients’ conception of pain has the potential to improve doctor-patient communication.

## Introduction

### Background

Pain is a very complex and subjective phenomenon. It is regarded as a symptom of an underlying condition or as a condition of its own. For adequate medical treatment, however, it is compulsory to classify the reported pain. Hints to the correct pain diagnosis are given by a pain assessment looking at the intensity of the pain, its distribution and duration, as well as the quality of the pain. Despite many new technological advances, however, objectification of pain is still an unsolved problem [1]. Asking patients to draw their pain has been used for half a century to overcome the complexity of communicating a subjective sensation from patient to physician. This method has different names in the literature, the most common being pain drawing (PD). Different instruments have been used to obtain PDs, starting from pen-on-paper drawings [2] and recently developing toward electronic PDs collected on tablet personal computers (PCs) [3-7]. Several studies have been conducted to test the usability, reliability, and repeatability of PDs in chronic pain situations such as shoulder pain [8], knee pain [9], back pain [10], and neck pain [11] as well as in acute low back pain [12-14], whiplash disorder [15], or experimentally triggered pain [16]. Regardless of the method used, it has been proven that using PDs together with anamnesis and physical examination can aid the differential diagnosis in many pain situations [9,10].

### Objectives

In this study, we examined the potential role of electronic PDs collected on a tablet PC in the clinical assessment and treatment of inpatients in acute pain situations. Therefore, we evaluated if knowing the patients’ PDs improved the pain specialists’ understanding of their patients and influenced their clinical decision making. Furthermore, we sought to identify differences between PDs of patients and doctors and to find those specific characteristics of the drawings that had the largest impact on doctors’ understanding.

To this end, we collected electronic PDs from a sample of inpatients that received a consultation by a pain specialist from acute pain service (APS) because of severe pain (average score of 7.3, SD 2.0 on an 11-point numeric rating scale [NRS]). We then asked the pain specialists to draw their own impression of the patients’ pain after anamnesis and physical examination but without having seen the patients’ drawings. PDs of the patients were then revealed to the doctors, and they were asked to evaluate how much the additional information improved their understanding of the case and how much it influenced their clinical decision. Similarities and differences of patients’ and doctors’ PDs were assessed by statistical image analysis and visual inspection. Finally, exploratory analyses were conducted to identify specific factors that had the greatest impact on doctors’ understanding of the patients.

## Methods

### Study Population

Our study population were inpatients from different departments of Hannover Medical School. All patients were in acute pain situations that required a consultation by a pain specialist, which is provided by members of the APS. APS members visit the patients and adjust their pain management individually according to the requirements of each patient’s situation. Eligible patients were adult (age ≥18 years in Germany) inpatients of Hannover Medical School with acute pain and the ability to give written informed consent. Furthermore, they had to be physically able to complete an electronic PD on a tablet PC. We recruited 69 patients (37 females) for participation in our study, and all of them prepared a PD. Due to a technical failure of the tablet PC, 1 drawing was lost. The treating pain specialist prepared a PD for 61 of the remaining patients, all of which were included in the analysis. Of these 61 patients with complete data, however, only 47 (24 females) were discussed and rated because of the absence of some of the treating pain specialists during the meeting of the APS in the afternoon (see below). Characteristics of the final study population can be found in Table 1.

### Procedures

The study was approved by the Ethics Committee of Hannover Medical School (#2987-2015) and was conducted in accordance with the Declaration of Helsinki. All patients gave written informed consent after information of the purpose of the study.

Data collection was divided into 3 steps: (1) the patient draws a PD shortly before the consultation, (2) the pain specialist draws a PD of the same patient directly after the consultation based on anamnesis and bodily examination results, and (3) the pain specialist rates the patient’s PD’s influence on his or her understanding of the patient as well as on clinical decision making.

#### Patients’ Pain Drawings

Two of the authors (NS and AA) screened all patients on schedule for consultation by the APS. All eligible patients were informed about the study purpose and asked for participation after checking inclusion criteria. Written informed consent was obtained, and patients were asked to rate their pain intensity on an 11-point NRS ranging from 0 for “no symptom” to 10 for “maximum imaginable intensity.” Next, the use of the tablet PC and the SymptomMapper app [17] was explained to the participant, and an electronic drawing of the acute pain and related sensations was acquired using the following instructions: “Please draw the location of your sensations as accurately as possible. Make sure to draw all sensations that you perceive as unpleasant or unnormal.” These instructions were complemented by a graphical depiction in the app’s drawing instructions module (see below), and patients were supervised by NS or AA during the drawing process.

**Table 1:** Demographics of our study population.

Characteristics		Statistics
Age in years, mean (SD)		59.2 (15.9)
**Age range in years, n (%)**		
	18-39	7 (15)
	40-59	15 (32)
	60-79	21 (45)
	80+	4 (9)
Women, n (%)		24 (51)
Numeric rating scale pain intensity, mean (SD)		7.3 (2.0)
**Origin of pain, n (%)**		
	Cancer	18 (38)
	Infection	8 (17)
	Postsurgical	5 (11)
	Neurological	3 (6)
	Other	13 (28)

#### Doctors’ Pain Drawings

Consultation by the APS consisted of anamnesis and clinical examination by a member of the service. All members participating in the study were anesthesiologists with at least 5 years of clinical experience and either pain specialists or in training for pain specialization. On the basis of the anamnesis and findings of the physical examination, doctors drew their patients’ pain and related sensations during or immediately after the consultation but before seeing the patients’ drawing. They used the same kind of tablet PC and app like the patients. However, in addition to pain and related sensations, they were also free to draw pain-related symptoms such as allodynia, hyperalgesia, erythema, swelling, hyperhidrosis, and muscular defense. After doctors had finished their PD, they continued with their usual clinical procedures, such as starting the pain treatment or modification of an ongoing treatment.

#### Rating of Knowledge Gained From Pain Drawings

Patients’ PDs were shown to the doctors during the meeting of the APS in the afternoon, where each new patient is reviewed and treatment options are discussed. When discussing a study participant, we revealed his or her PD to all doctors, and they were free to discuss it. After the meeting, the doctors were asked for their anonymous rating of the following 2 questions: (1) “How much did the electronic PD improve your understanding of the patient?” and (2) “How much did the electronic PD influence your clinical decision?” Both questions were followed by an 11-point Likert scale from 0 for “not at all” to 10 for “very much.” Doctors were only allowed to rate PDs from patients that they themselves were treating.

### Data Acquisition

#### Tablet Computer

All electronic PDs were acquired on tablet PCs type Samsung Galaxy Note 2014 edition 10.1 (SM-P600) running Android 5.1.1 with an electronic pen (stylus) based on inductive digitizing technology. The tablet had a 10.1-inch touch screen with a resolution of 800×1280 pixels, and its stylus was used for all data entry. In contrast to entering data by finger on the capacitive touchscreen, the tablet records stylus interactions with a separate inductive digitizer, which allows for a higher resolution while eliminating unwanted activation of the screen, for example, by the palm.

#### Software App

**Figure 1:**
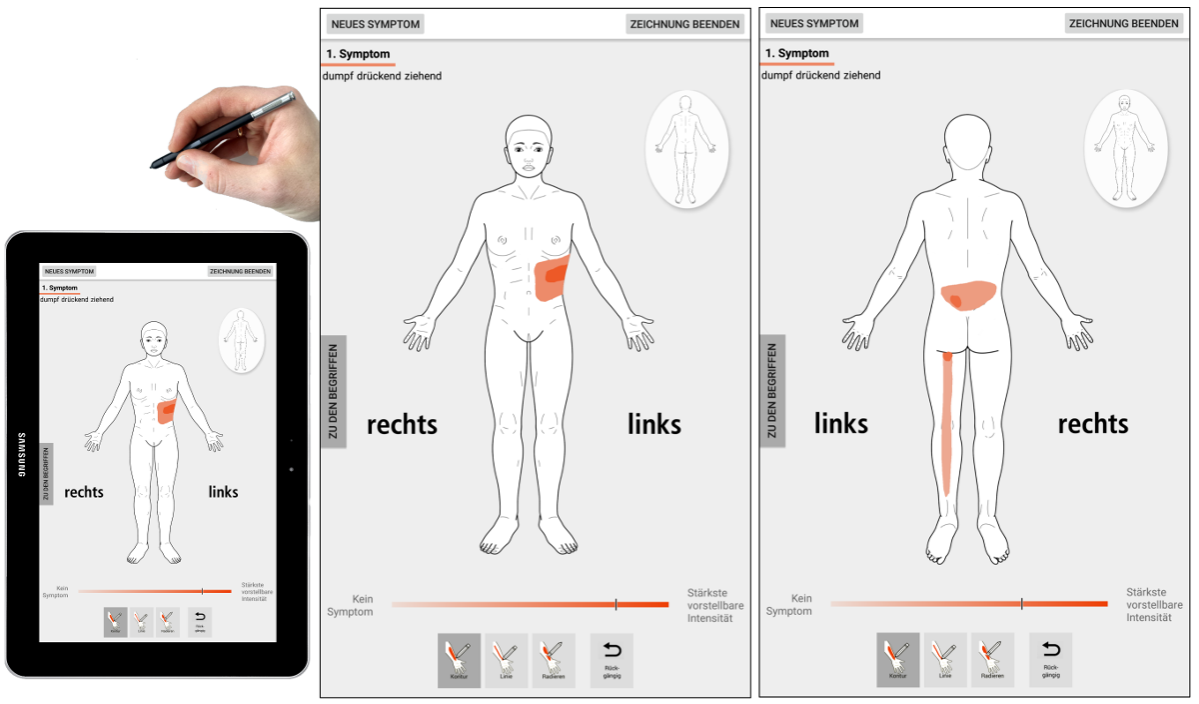
Graphical user interface of the SymptomMapper app that was used in our study. Its drawing module allows for quick and easy data entry without previous training, a crucial prerequisite when studying patients in acute pain situations. Sides are emphasized by the words left (“links”) and right (“rechts”). Doctors and patients used the same app for their pain drawings.

We used SymptomMapper [17], a software app developed by our group to acquire the PDs from both patients and doctors (see Figure 1). The app consisted of 3 different modules: drawing instructions, symptom specification, and drawing. App versions for patients and doctors used the same modules but with slightly different content. The average time to complete a drawing ranges from 1 to 10 min depending on the level of details and number of symptoms drawn. We have previously shown that SymptomMapper has a good usability for patients and doctors and that test-retest reliability of symptom drawings by chronic pain patients have fair reproducibility for the exact symptom pattern but excellent reproducibility for symptom extent [17].

In the symptom specification module, users were asked to specify any pain-related symptom in an iterative process. They first chose the type of sensation from the following list of descriptors (in German): burning, cold, cramping, dull, electric, heavy, hot, numb, pressing, pricking, shooting, stabbing, tender, throbbing, tingling, and tugging. Next, they rated the intensity of the sensation on a visual analog scale, ranging from “no symptom” to “strongest imaginable intensity.” Finally, they entered the perceived depth of the sensations by choosing among different depth descriptors.

In the drawing instructions module, the user was asked to color every point of the body outline where the specified pain sensation or related symptom was present and to use all available body views. Other ways to mark a body region, such as hatching, ticking, or marking by arrows or other symbols, were explicitly prohibited.

In the final module, users were shown a body outline of matched sex to draw the location of the symptom or finding specified in the previous module. Drawings could only be made using the tablet’s stylus, and drawing was restricted by the app to within the borders of the body outline. Adherence to the other drawing instructions was checked by the author supervising the drawing process (NS or AA). After finishing the drawing, users could either choose to end data entry or to add another symptom, which would bring them back to symptom specification.

The doctors’ version of the app differed only slightly from the patients’, in that its symptom specification module provided the user with a list of common pain-related diagnostic findings in addition to the list of pain descriptors.

### Data Analysis

#### Statistical Analysis

All statistical calculations were done in R version 3.4.3 (R Foundation for Statistical Computing, Vienna, Austria) [18] and Microsoft Excel (Microsoft Corporation, Redmont, WA) using the Real Statistics Resource Pack release 5.4.1 [19]. PDs were converted from Portable Network Graphics format to Neuroimaging Informatics Technology Initiative format [20], with a custom-written Python script (Python 2.7, Python Software Foundation [21]), and analyzed using FMRIB Software Library (FSL) version 5.0 (FMRIB Analysis Group, Oxford University, UK) [22]. Final figures were prepared using VINCI 4.86.0 (Max Planck Institute for Metabolism Research, Cologne, Germany) [23] and GNU Image Manipulation Program version 2.8.16 (GIMP, The GIMP Team) [24].

#### Impact on Understanding of Pain and Clinical Decision

The average of doctors’ ratings of improvement in understanding and influence on clinical decision were individually tested for their difference from zero using a 1-sided 1-sample *t* test. Here and in all further statistical tests, a *P* value of .05 or less was considered significant.

#### Pain Drawing Analysis

We extracted the following data from each PD for statistical analysis: (1) number of drawn pixels (pain extent), (2) number of clusters, (3) number of body views used in PD, (4) number of symptom descriptors, (5) average intensity per symptom, and (6) widespread pain index [25]. The first 2 quantities were also calculated for images thresholded at pain intensity larger or equal to 6 to focus on the most severe symptoms. The pain extent (thresholded and unthresholded) was normalized to percent template surface for each view by dividing the pixel count by the total number of pixels of the respective view of the body outline. Widespread pain index (WPI) was calculated by masking the PD with a custom-made template of the 19 body regions used in the WPI and counting the number of nonempty body regions. Please note that signs and symptoms other than pain or paresthesia as recorded by the doctors’ version of the app were not included in the PD analysis.

#### Comparison of Patients’ and Doctors’ Pain Drawings

To identify potential systematic differences between doctors’ and patients’ PDs, we calculated 2-sided paired *t* tests for all quantities mentioned above. Furthermore, we assessed the similarity of doctors’ and patients’ PDs by means of the Jaccard index and by calculating intraclass correlation coefficients (ICCs (3,1); Shrout and Fleiss classification) for the pain area and the number of symptom clusters. Both indices were calculated separately for each body view and averaged over all body views excluding those that were empty in both the doctors’ and the patients’ PD. When the drawing contained multiple pain symptoms, they were merged, and the maximum intensity was used for each pixel.

We further calculated the average pain distribution of all patients for visual comparison by pixel-wise averaging of all these individual drawings using FSL Maths. Doctors’ and patients’ PDs were analyzed independently. Due to the large diversity of pain states and syndromes encountered in our study sample, we did not attempt a direct statistical comparison of the data. The final images were thresholded to show only those body regions where at least 10% (5/47) of all users had drawn a symptom. This was an arbitrary threshold used to reduce the impact of single drawings with very large pain areas. As doctors’ and patients’ drawings were thresholded the same way, it does not obscure any differences between the 2 but allows the reader to focus on relevant areas.

#### Exploratory Analysis of Relevant Factors

To identify factors of relevance that improved doctors’ understanding of their patients’ pain, we calculated cross- correlation coefficients for the ratings of improvement in understanding and the quantities derived from the patients’ PDs (see above).

## Results

### Impact on Understanding of Pain and Clinical Decision

**Figure 2:**
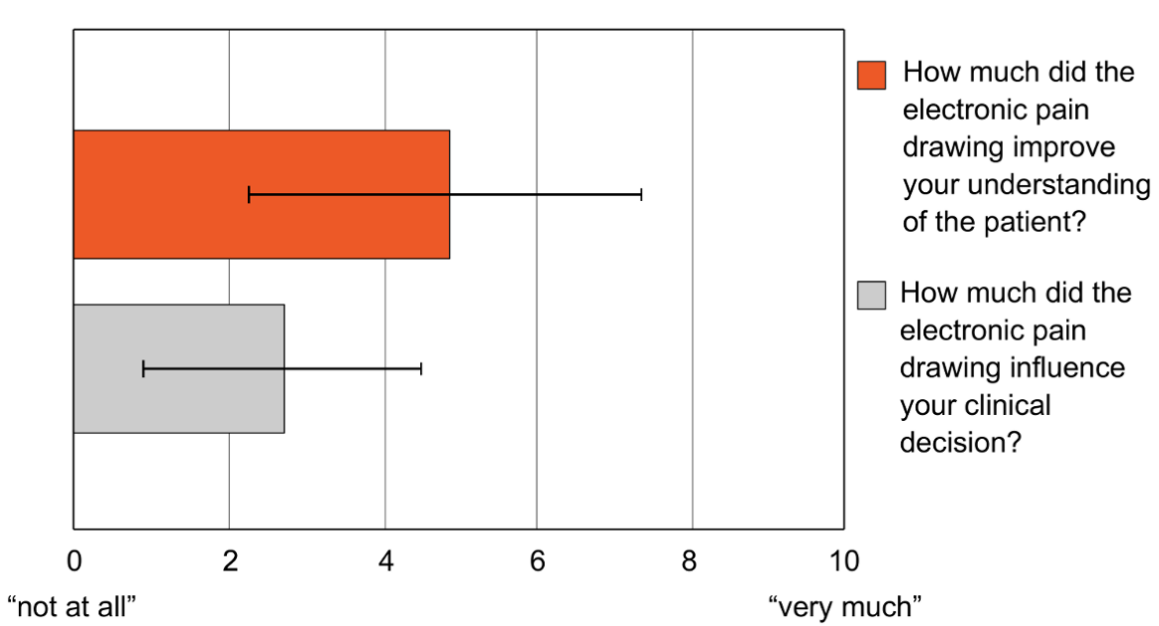
Impact of knowing patients’ pain drawings (PDs) on understanding of the pain and clinical decision making as rated by the doctors. Patients’ PDs significantly improved the doctors’ understanding of the pain and to a lesser but still significant extent influenced their clinical decision.

Knowing patients’ PDs significantly improved the doctors’ understanding of their patients (average rating: 4.81, SD 2.60, *P*<.001) and to a lesser extent their clinical decision (average rating: 2.68, SD 1.18, *P*<.001). Results are shown in Figure 2.

### Comparison of Patients’ and Doctors’ Drawings

**Figure 3:**
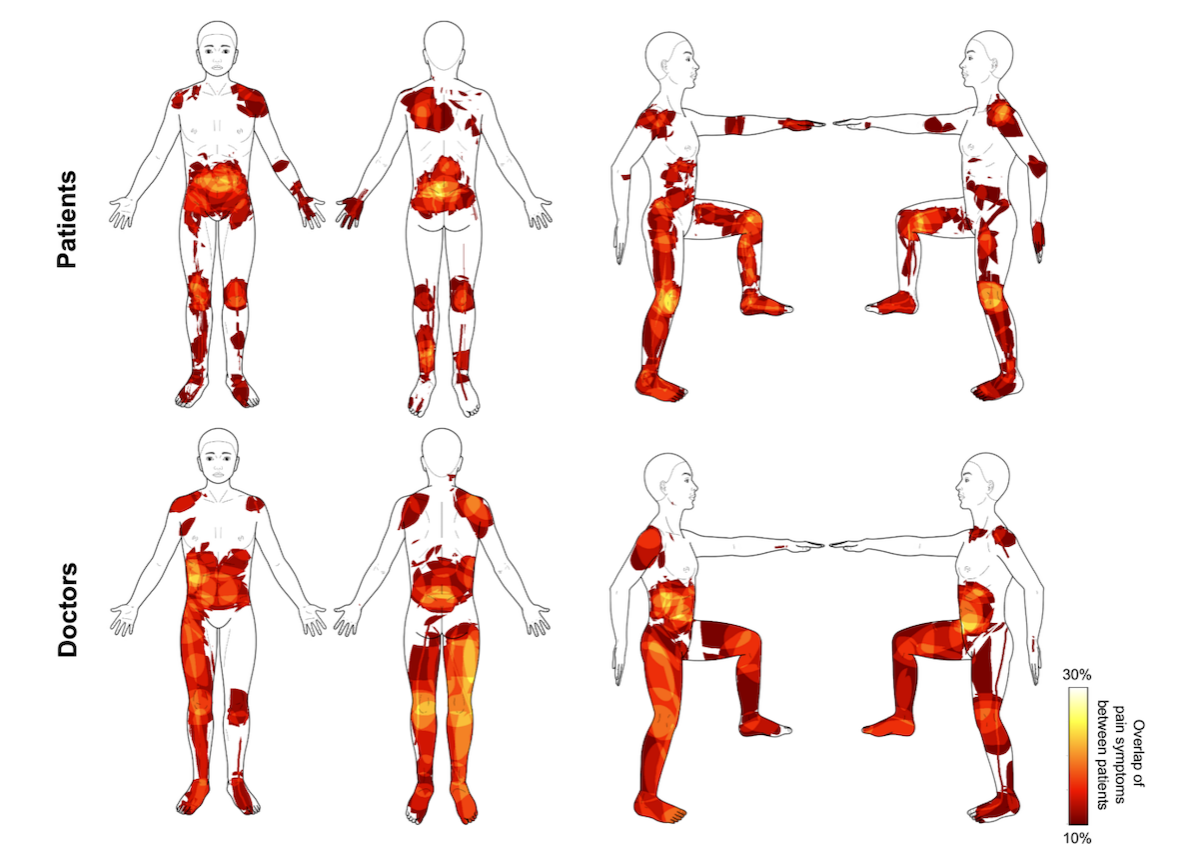
Descriptive comparison of patients’ (top line) and doctors’ (lower line) perception of pain in our final sample of 47 acute pain patients. Average pain distribution thresholded at 10% overlap between patients.

Patients drew on average 1.25 (SD 0.53) pain symptoms, a number closely matched by the doctors’ who drew 1.34 (SD 0.64) symptoms. With 3.34 (SD 2.82) different pain descriptors, patients described their pain more detailed (*P*=.03) than the doctors, who used 2.43 (SD 1.30) descriptors. The average pain distribution drawn by patients and doctors and the frequencies of pain descriptors are shown in Figure 3 and Table 2, respectively. Visual comparison of the averaged PDs suggested a high similarity of doctors’ and patients’ drawings. Similarity analysis of the individual PDs revealed fair reproducibility for pain extent with an ICC of 0.565 (95% CI 0.459-0.655) but poor reproducibility for the number of pain clusters with an ICC of 0.368 (95% CI 0.238-0.485). The Jaccard index was 0.217 (SD 0.171). Detailed results for each body view are listed in Table 3. The poor reproducibility of the number of pain clusters also showed when we compared PD characteristics directly between the 2 groups (Table 4). Here, we found that patients drew significantly more pain clusters when comparing unthresholded (*P*<.001) and thresholded clusters (*P*=.01). Pain extent, average pain intensity, and the number of nonempty body views on the other hand showed no significant differences between patients and doctors.

**Table 2:** Frequency of symptom descriptors.

Symptom descriptor	Patients, n	Doctors, n
Stinging	22	28
Burning	18	16
Pressing	16	15
Tugging	15	11
Radiating	13	6
Dull	10	7
Cramping	11	5
Tingling	10	4
Shooting	5	8
Electric	4	7
Heavy	7	1
Tender	6	2
Throbbing	7	1
Pricking	4	2
Numb	4	1
Hot	4	0
Cold	1	0
Total	157	114

**Table 3:** Similarity of doctors’ and patients’ pain drawings.

Analysis			Result
Jaccard index of symptom pattern, mean (SD)			0.22 (0.17)
**ICC^a^** **of symptom extent (95% CI)**			
	Whole drawing (all body views)		0.57 (0.46-0.66)
	**Single views**		
		Front	0.51 (0.26-0.69)
		Back	0.52 (0.28-0.70)
		Left	0.56 (0.32-0.73)
		Right	0.70 (0.51- 0.82)
**ICC of number of symptom clusters (95% CI)**			
	Whole drawing (all body views)		0.37 (0.24-0.49)
	**Single views**		
		Front	0.32 (0.04-0.55)
		Back	0.33 (0.05-0.56)
		Left	0.42 (0.15-0.63)
		Right	0.43 (0.17-0.64)

^a^ICC: intraclass correlation coefficient.

**Table 4:** Comparison of doctors’ and patients’ pain drawing characteristics.

Pain drawings characteristics	Patients, mean (SD)	Doctors, mean (SD)	*P* value^a^
Pain extent^b^	7.08 (9.66)	8.12 (14.13)	.55
Pain extent (Visual Analog Scale >6)	5.69 (9.51)	7.15 (14.03)	.39
Number of pain clusters	3.63 (3.23)	1.81 (1.33)	<.001
Number of pain clusters (Visual Analog Scale >6)	2.59 (3.18)	1.48 (1.33)	.01
Number of nonempty body views	3.40 (0.74)	3.30 (0.95)	.40
Total number of symptom descriptors	3.34 (2.82)	2.43 (1.30)	.03
Average pain intensity	7.19 (2.17)	7.46 (1.82)	.33

^a^Paired 2-tailed *t* test.

^b^In percent template surface.

**Figure 4:**
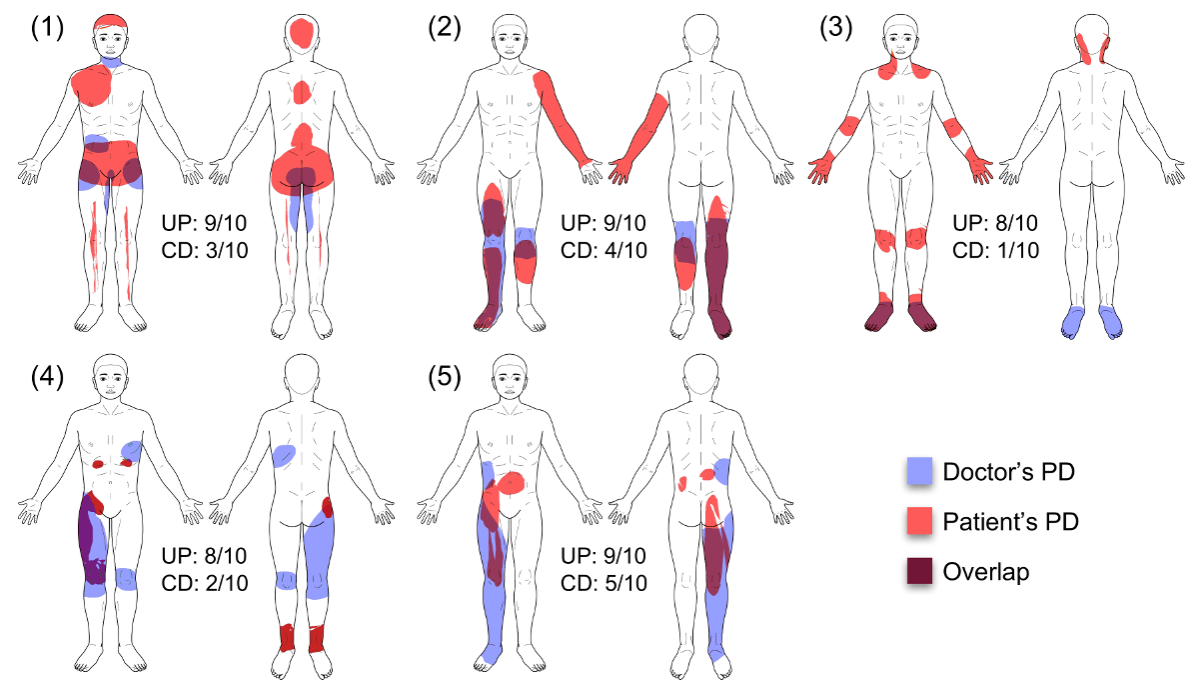
A comparison of patients’ and doctors’ pain drawings (PDs) for individual patients, in which knowledge of the PD led to strong improvement of the doctor’s understanding of the patient. CD: impact on clinical decision; UP: understanding of the patient.

### Patient Characteristics of Representative Cases

We identified 5 patients who received either very low or very high ratings from the doctors, that is, patients in which seeing the PD was of very little or very high value for the doctors understanding. The group with high ratings is shown in Figure 4, the group with the low ratings in Multimedia Appendix 1. The individual clinical cases are discussed in Table 5.

### Exploratory Analysis of Relevant Factors

**Figure 5:**
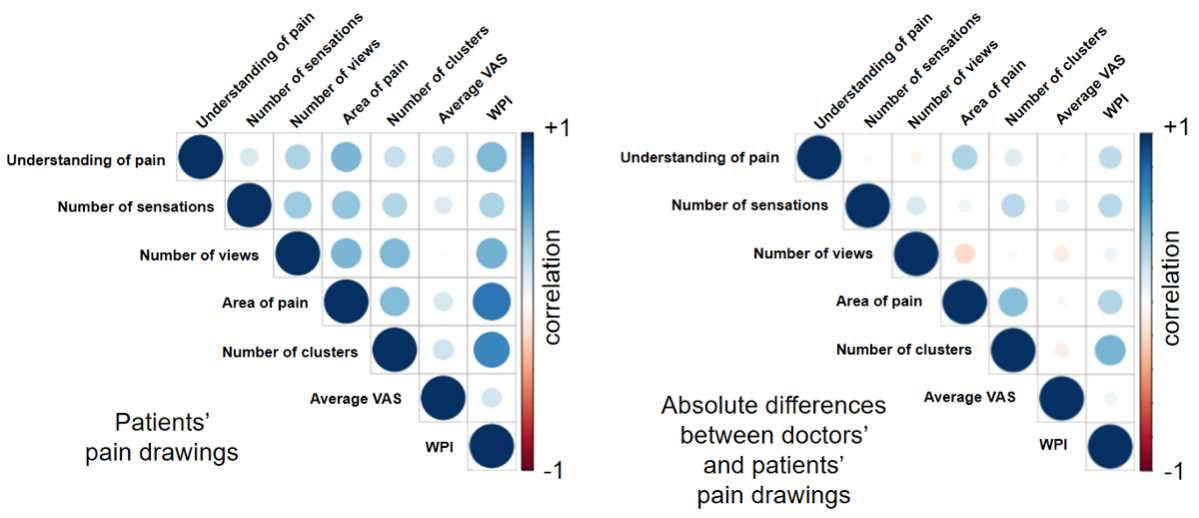
Factors with the potential to influence doctors’ understanding of the patients. The left image shows correlations of pain drawing characteristics extracted from the patients’ drawings, whereas the right image is based on absolute differences of those characteristics between patients’ and doctors’ drawings. Correlation strength is encoded in color brightness and circle size. Blue color indicates positive values and red color indicates negative values. Both the areas of pain (in percent body area) and the widespread pain index (WPI) showed significant correlations with the doctors’ understanding of pain. VAS: visual analog scale.

Exploratory cross-correlation analysis revealed that the understanding of pain was influenced most strongly by 2 factors: the area of the pain as drawn by the patient (*r*=.454, *P*=.001) and the WPI (*r*=.447, *P*=.001) as calculated from the PD (see Figure 5). In both cases, the correlation was positive, which means that a larger pain area and higher WPI were associated with greater improvement in understanding of pain. When testing the same factors but looking at their absolute differences in doctors’ and patients’ drawings, area of pain showed the only significant correlation with understanding of pain (*r*=.313, *P*=.03), whereas WPI showed a tendency (*r*=.255, *P*=.08).

**Table 5:** Discussion of the patients in which knowledge of the PD led to strong improvement of the doctor’s understanding of them.

Patient		Description
**Patient 1** **(female, 45 years)**		
	Indication for hospital admission	Unexplained abdominal pain
	Indication for presentation to APS^a^	Severe abdominal pain
	Diagnosis	Somatization disorder
	History	Diagnostic laparoscopy (10 weeks before admission) and hysterectomy (3 years ago)
	Notes	Pain cluster in the neck appeared after laparoscopy and can be explained by irritation upon endotracheal intubation
	Knowledge gained from patient’s PD^b^	Additional pain clusters in the patient’s PD supported the clinical diagnosis of somatization disorder
	Implications for treatment	Referral to further psychiatric and psychosomatic treatment; discontinuation of antinociceptive therapy
**Patient 2** **(male, 63 years)**		
	Indication for hospital admission	Surgery: transcatheter aortic valve implantation for aortic stenosis
	Indication for presentation to APS	Acute pain in the right leg
	Diagnosis	Exacerbation of pain in the right leg with mixed nociceptive, ischemic, and neuropathic pain states in the course of peripheral arterial occlusive disease
	History	Transtibial amputation of the left leg; pain syndrome of the cervical spine
	Notes	No phantom limb pain in the left leg; pain cluster in the left arm and hand can be explained by pre-existing pain syndrome of the cervical spine
	Knowledge gained from patient’s PD	Comprehensive overview of pain clusters originating from different causes
	Implications for treatment	None
**Patient 3** **(female, 36 years)**		
	Indication for hospital admission	Surgery: cyclophotocoagulation status post chronic open-angle glaucoma
	Indication for presentation to APS	Acute pain in both feet
	Diagnosis	Exacerbation of pre-existing pain in both feet from polyneuropathy in the course of Wegener granulomatosis
	History	Wegener granulomatosis with joint involvement; polyneuropathy
	Knowledge gained from patient’s PD	Additional pain clusters in the patients’ PD supported the clinical understanding of the widespread manifestations of the underlying disease
	Implications for treatment	Referral to specialized outpatient pain treatment
**Patient 4** **(male, 80 years)**		
	Indication for hospital admission	Acute pain exacerbation with suspicion of cancer
	Indication for presentation to APS	Acute pain in the right upper limb, right knee, and costal arch
	Diagnosis	Exacerbation of pre-existing pain due to because of multiple cancerous osteolytic lesions from unknown primary
	History	Pre-existing pain in the abovementioned regions starting 3 to 1 weeks before admission
	Knowledge gained from patient’s PD	Comprehensive overview of all pain sites
**Patient 5** **(male, 82 years)**		
	Indication for hospital admission	Urinary tract infection and deterioration of the patient’s general condition
	Indication for presentation to APS	Acute pain in the right leg and flank
	Diagnosis	Exacerbation of 2 different pre-existing pain states; neuropathic pain in the right leg; visceral pain in the area of the right kidney
	History	Urothelial carcinoma (UICC-Classification (Union for International Cancer Control-Classification) pTx, pNx, G3, L1, V1) and recurrent urinary tract infections under treatment with a double-J catheter; pre-existing pain in the abovementioned regions starting 3 to 1 months before admission
	Knowledge gained from patient’s PD	Comprehensive overview of pain clusters originating from different causes; pain pattern confirmed the neuropathic origin of the pain in the leg
	Implications for treatment	Start of an antineuropathic treatment

^a^APS: acute pain service.

^b^PD: pain drawing.

## Discussion

### Overview

In this study, we have tested whether knowledge of the electronic PD of a patient in acute pain can improve the doctors’ understanding of the patient’s pain and potentially influence clinical decision making. We have furthermore sought to identify similarities and differences of electronic PDs made by patients and their treating pain specialists. Finally, we wanted to find specific characteristics of the drawings that have a large impact on doctors’ understanding.

### Impact on Understanding of Pain and Clinical Decision

Our results show that PDs significantly improved the doctors’ understanding of their patients’ pain based on their own judgment. On average, doctors rated this improvement with 4.81 out of 10 points. The impact on clinical decision was also significant but of smaller size (2.68 out of 10 points). There are 2 possible explanations for the fact that a relatively large improvement in understanding resulted in rather modest changes in clinical decision. First, in the majority of cases, additional pain clusters drawn by the patients do not lead the physicians to new diagnoses that would require additional medical investigation or intervention. Instead, most of these clusters reveal previously diagnosed chronic pain sources that are unrelated to the acute problem. To take one of our examples (patient number 2 from Figure 4 and Table 5), a patient requiring consultation by the APS for acute leg pain from peripheral arterial occlusive disease draws pain clusters in the leg region but also adds a large cluster in the arm. The latter stems from a previously diagnosed pain syndrome of the cervical spine. Although this additional knowledge gives a more complete image of the patient and, therefore, improves the doctor’s understanding, it has little impact on the clinical decision.

### Comparison of Patients’ and Doctors’ Drawings

The similarity of PDs from patients and their treating doctors observed in our study was considerably lower compared with studies looking at test-retest reliability of PDs in chronic pain patients. We found only fair reproducibility for pain extent (ICC: 0.57) and poor reproducibility for the number of pain clusters (ICC: 0.37). In contrast, Barbero et al and Neubert et al, using PDs from different chronic pain populations, found ICCs from 0.92 to 0.97 for pain extent [3,17]. Results regarding the reproducibility of the number of pain clusters differed even more. Here, Neubert et al report an ICC of 0.70, which is almost twice as high as the value found in our study. The Jaccard index of 0.22 (as compared with 0.46-0.49 in the above-mentioned studies) indicates an average overlap of only 22% between the PD of a patient and the associated drawing of the doctor in our study. Of course, we are comparing apples with pears here, as repeated PDs by the same person will be much more similar than PDs based on information derived from verbal and nonverbal communication. A comparison like this, however, allows us to get an estimate for how much information is lost or changed in patient-doctor communication.

### Which Drawing Is “Correct”?

The above-mentioned issue raises the rather philosophical question, which of the drawings contains the correct information. On the one hand, only the patient is able to perceive the symptoms that are expressed in the PD. As several studies have shown, generating a PD is a highly reproducible and reliable process [15,16]. Thus, there are good reasons to argue that patients’ PDs constitute the ground truth in this comparison. However, other studies have based their analysis on the assumption that the doctors’ drawings contain the correct information. For example, Cummings et al compared patients’ PDs with drawings by their doctors and found that the usage of patients’ PDs to measure pain extent, number of pain clusters, and related symptoms may lead to inaccurate diagnoses [26]. It should also be noted that anamnesis and physical examination themselves can lead to more detailed PDs by the doctors as they may reveal additional symptoms omitted by the patient.

In our opinion, both patients’ and doctors’ PDs are valid drawings in their own right. When analyzing their contents, however, it must be acknowledged that they do not represent the same thing and that there may be systematic differences that will influence the results of the analysis. These differences will be discussed in the next section.

### Potential Sources of Systematic Differences

In our study, we did not find significant differences between patients’ and doctors’ PDs regarding pain extent, average pain intensity, and the number of body views used in for the PD. Together with our findings regarding the low similarity of doctors’ and patients’ drawings, this means that both groups drew about the same number of pixels with the same intensity but in different places on the body. We further found that doctors used significantly fewer pain clusters and symptom descriptors than patients when drawing their pain symptoms. There are several possible explanations for this. On the one hand, it is likely that doctors’ drawings exhibit less specificity of pain location than the patients’, simply because some level of information loss is to be expected for verbal communication of bodily symptoms. This can be seen in Multimedia Appendix 1, where clusters drawn by doctors are clearly overlapping with those of the patients but are generally larger and, thus, less specific. On the other hand, doctors may have focused more on single symptoms with larger extent. This could be explained by the principles of the APS [27]. In contrast to outpatient care settings, inpatients are well diagnosed and known to their treating medical team. Therefore, these patients are presented with a specific question to the specialists of the APS. Pre-existing and already treated pain diagnoses are not as much in the focus of interest as they would be in an outpatient setting.

### Pain Drawing Characteristics That Can Improve the Understanding

Our innovative methodology of electronic PD analysis allowed us to extract a variety of information from the acquired PDs. This included characteristics such as the area of pain, average intensity, and WPI [25] as well as the number of clusters, pain sensations, and body views used in the drawing. Availability of this information enabled us to perform an exploratory analysis to identify those characteristics that significantly improved doctors’ understanding of their patients’ pain. We found that both pain area and WPI had the largest impact on doctors’ understanding. Thus, not only drawings with more pain area received higher ratings but also those where the pain was more widespread. Both effects can be observed when comparing patients with the highest ratings (Figure 4) with those with the lowest (Multimedia Appendix 1). It is evident that the latter show much smaller pain areas and less widespreadness than the former. However, pain area and WPI also showed a high level of correlation with each other, indicating similar information content.

When looking at differences between patients’ and doctors’ PDs, however, only the absolute differences in pain area improved the doctors’ understanding. Thus, an over- or underestimation of pain area by the doctor made seeing the patient’s PD valuable for the doctor. Among other things, this finding indicates that doctors do consider information from the patients’ PD as being “correct.”

### Advantages of Electronic Pain Drawings

Although our study was not aimed at comparing electronic PDs with their pen-on-paper counterparts, we would nevertheless like to emphasize the advantages of using the electronic version. In the last 10 years, several research groups have developed PD apps to be used on tablet computers [3-7,28]. A central advantage of the electronic drawings acquired this way is the possibility to analyze results right after completion of the drawings and without the need for time-consuming digitization. Although measuring pain area is possible for pain-on-paper drawings by using grid-based methods [13,29], such analyses usually take several minutes and require the doctor to sit down at a table with adequate lighting. Furthermore, the calculation of more complicated but relevant variables, such as average pain intensity or pain overlap as used in our paper, would take even longer to extract from pen-on-paper drawings. Finally, well-designed PD apps allow the user to zoom in and, thus, can be used by people with severe visual impairments for whom conventional drawings would be challenging or even impossible.

### Limitations

Although our study has reached the planned aims, there were some limitations that we could not avoid. First, our sample consisted of inpatients in acute and often severe pain. Thus, the accuracy of completing PDs may have been lower compared with, for example, chronic pain patients that have had some time to adapt to their pain. Of course, such lower accuracy will also influence all further analyses, for example, regarding similarity of patients’ and doctors’ PDs. Second, our procedure of rating the improvement in understanding and influence on clinical decision was suboptimal as only the treating doctor was allowed to give a rating and this rating was anonymous. This made it impossible to assess potential bias, for example, by certain doctors, giving only good or bad ratings. Furthermore, we did not ask for the explicit reasons why a PD was considered helpful or why its knowledge did or did not influence clinical decision. Third, our sample size of 47 patients was rather small and may have led to false-negative results in all analyses directly comparing patients with doctors. Finally, the fact that all doctors that rated the impact of the patients’ drawings on their understanding and clinical decision also prepared drawings themselves may be seen as a confound. Although we believe that the instructions for rating were unmistakably aimed at the impact of seeing the patients’ drawing (and not, eg, of comparing the patients with their own drawing), we cannot rule out the possibility that the act of drawing may have confounded the rating in some individuals.

Future studies should assess the importance of patients’ PDs independently from that of PDs made by the doctors, that is, compare doctors using the app with those not using it. Furthermore, it would be desirable to assess improved understanding of the patient by more objective means than self-report.

### Conclusions

We have shown that in a clinical setting, electronic PD can improve doctors’ understanding of patients in acute pain situations based on their own judgment. The ability of electronic PDs to visualize differences between doctors’ and patients’ conception of pain has the potential to improve doctor-patient communication.

## Appendix

Multimedia Appendix 1 A comparison of patients’ and doctors’ pain drawings (PDs) for individual patients, in which knowledge of the PDs led to small or no improvement of the doctor’s understanding of the patient.

## Abbreviations

APS: acute pain service
FSL: FMRIB Software Library
ICC: intraclass correlation coefficient
NRS: numeric rating scale
PC: personal computer
PD: pain drawing
WPI: widespread pain index
