# Figure Correction: Digital Pain Drawings Can Improve Doctors’ Understanding of Acute Pain Patients: Survey and Pain Drawing Analysis

**DOI:** 10.2196/16017

**Published:** 2019-09-27

**Authors:** Nour Shaballout, Anas Aloumar, Till-Ansgar Neubert, Martin Dusch, Florian Beissner

**Affiliations:** 1 Somatosensory and Autonomic Therapy Research Institute for Diagnostic and Interventional Neuroradiology Hannover Medical School Hannover Germany; 2 Section Pain Medicine Clinic of Anaesthesiology and Intensive Care Medicine Hannover Medical School Hannover Germany

In “Digital Pain Drawings Can Improve Doctors’ Understanding of Acute Pain Patients: Survey and Pain Drawing Analysis” by Shaballout et al (JMIR Mhealth Uhealth 2019;7(1):e11412), the authors inadvertently reversed the legends in the bar plot of Figure 2.

A revised version of Figure 2 has been uploaded with the correct legend wherein the upper (red) bar is denoted by “How much did the electronic pain drawing improve your understanding of the patient?” and the lower (grey) bar is denoted by “How much did the electronic pain drawing influence your clinical decision?”

The correction will appear in the online version of the paper on the JMIR website on September 27, 2019, together with the publication of this correction notice. Because this was made after submission to PubMed, PubMed Central, and other full-text repositories, the corrected article has also been resubmitted to those repositories.

**Figure 2:**
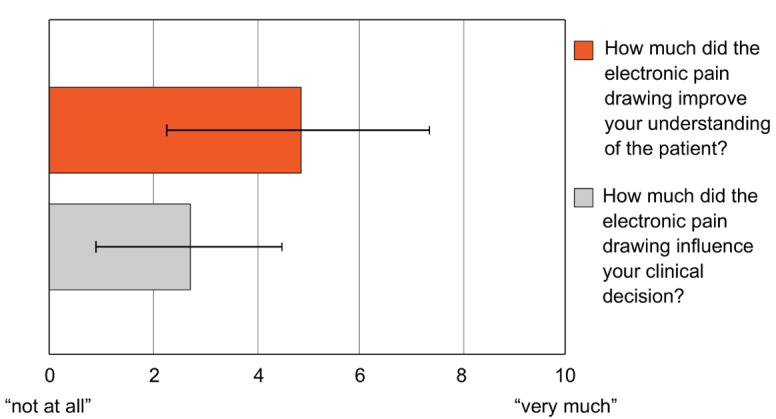
Impact of knowing patients’ pain drawings (PDs) on understanding of the pain and clinical decision making as rated by the doctors. Patients’ PDs significantly improved the doctors’ understanding of the pain and to a lesser but still significant extent influenced their clinical decision.

