# Supplementary figures and images for: Digital Pain Drawings Can Improve Doctors’ Understanding of Acute Pain Patients: Survey and Pain Drawing Analysis
Source: JMIR Mhealth Uhealth. 2019 Jan 10;7(1):e11412. doi: 10.2196/11412 (PMC6329897; doi:10.2196/11412)

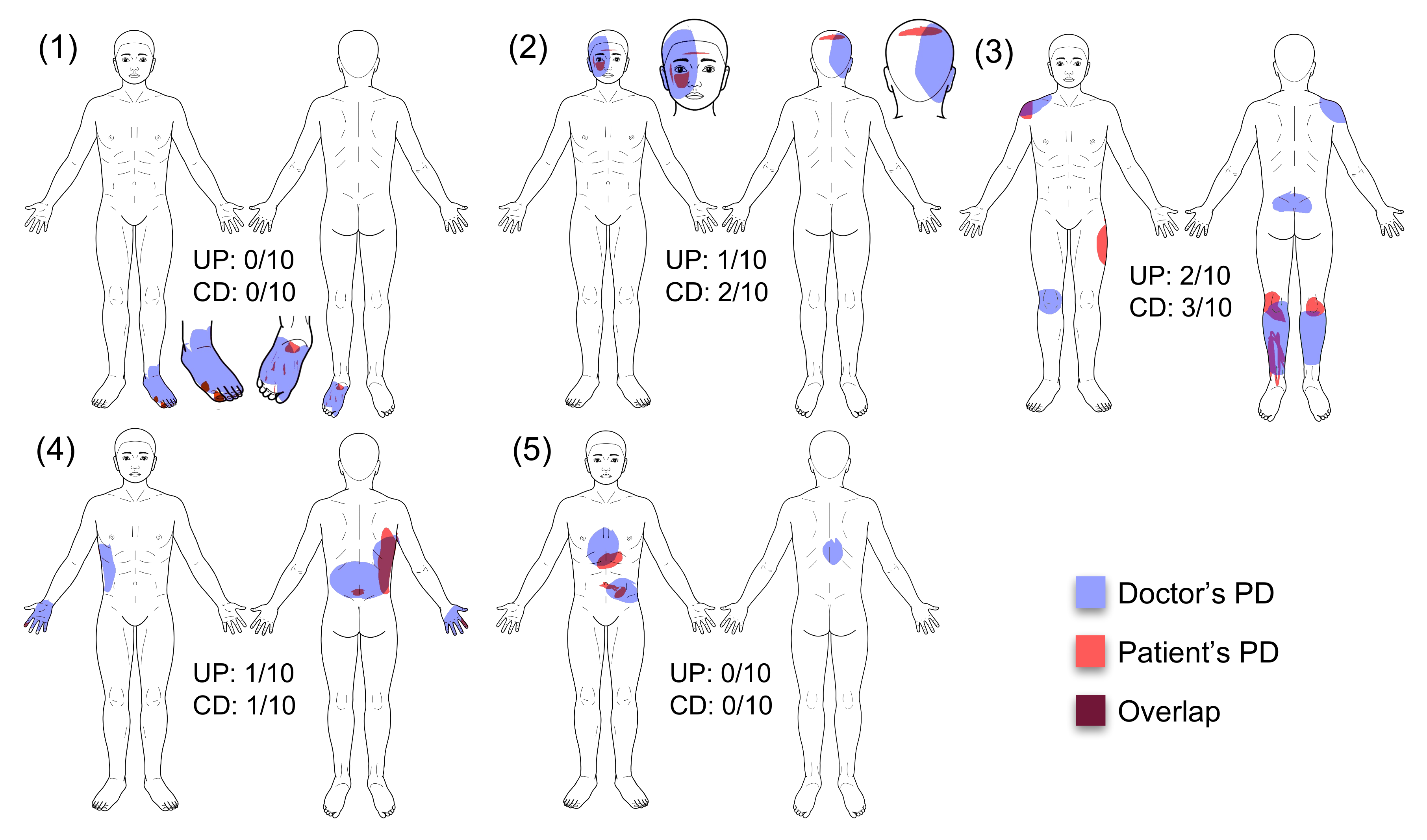

Supplement: Multimedia Appendix 1 [file mhealth_v7i1e11412_app1.png]
